# A case report of successful primary percutaneous coronary intervention to an occluded anomalous left main coronary artery arising from the right coronary sinus

**DOI:** 10.1093/ehjcr/ytae192

**Published:** 2024-04-15

**Authors:** Christopher C Y Wong, Brian P Pogatchnik, Daniel E Clark, Rahul P Sharma

**Affiliations:** Division of Cardiovascular Medicine, Stanford University School of Medicine, Stanford, CA, USA; Department of Radiology, Stanford University School of Medicine, Stanford, CA, USA; Division of Cardiovascular Medicine, Stanford University School of Medicine, Stanford, CA, USA; Division of Cardiovascular Medicine, Stanford University School of Medicine, Stanford, CA, USA

**Keywords:** Myocardial infarction, Percutaneous coronary intervention, Coronary vessel anomaly, Computed tomography, Case report

## Abstract

**Background:**

Anomalous aortic origin of a coronary artery from the opposite sinus is a rare congenital abnormality that may be encountered during primary percutaneous coronary intervention (PCI) for ST-elevation myocardial infarction (STEMI).

**Case summary:**

A 65-year-old man presented with chest pain and signs of heart failure. Electrocardiogram demonstrated atrial fibrillation with ST elevation in the high lateral leads, and he was taken emergently to the cardiac catheterization laboratory for primary PCI. Coronary angiography identified the culprit to be an occluded anomalous left main coronary artery (LMCA) arising from the right coronary cusp, and primary PCI was successfully performed in the LMCA and the left anterior descending artery (LAD). Computed tomography angiography confirmed a benign retroaortic course of the anomalous LMCA with no additional high-risk features, as well as a new left atrial appendage thrombus. He subsequently developed deep venous thrombosis, acute pulmonary embolism, and acute kidney injury secondary to renal artery embolism with associated infarction. Workup for patent foramen ovale and thrombophilia were negative, and he was discharged in a stable condition. At 2-month follow-up, he was asymptomatic with no evidence of myocardial ischaemia on stress cardiac magnetic resonance imaging.

**Discussion:**

We present the first reported case of an occluded anomalous LMCA arising from the right coronary sinus in a patient presenting with STEMI. Rapid recognition of this congenital anomaly and selection of an appropriate guide catheter were keys to achieving timely reperfusion and a good outcome in this case.

**Learning points**

- Clinicians should be cognizant of the possibility of anomalous aortic origin of a coronary artery in ST-elevation myocardial infarction patients when standard guide catheters fail to engage the coronary arteries.
- Computed tomography angiography is vital in delineating the course of the anomalous vessel, identifying high-risk features, and guiding further management in these patients.

## Introduction

Anomalous aortic origin of a coronary artery (AAOCA) from the opposite sinus is a rare congenital abnormality.^1^ The condition increases the technical complexity of percutaneous coronary intervention (PCI),^2,3^ particularly in patients with ST-elevation myocardial infarction (STEMI) in whom rapid reperfusion is critical to achieving an optimal outcome. In this report, we present the first known case of successful primary PCI in an occluded anomalous left main coronary artery (LMCA) arising from the right coronary sinus.

## Summary figure

**Figure:**
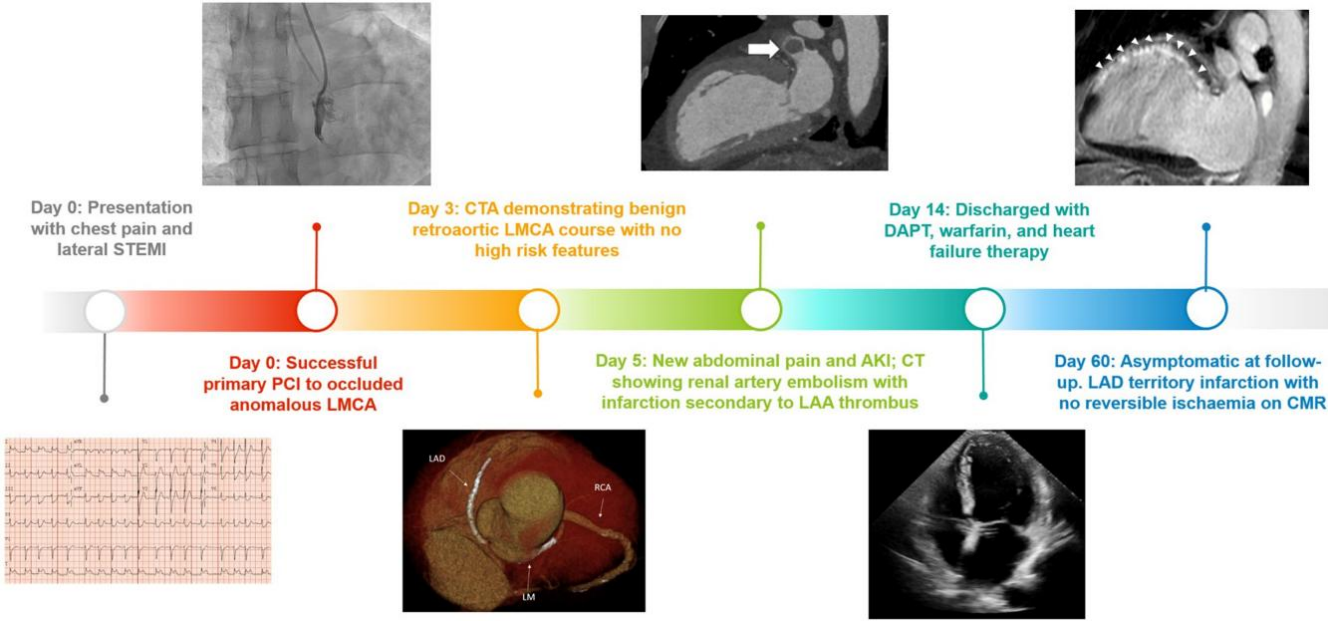


## Case presentation

A 65-year-old man presented to our hospital with sudden onset retrosternal chest pain that occurred while riding his bicycle uphill. On examination, he had a blood pressure of 168/83 mmHg, irregular pulse rate of 108 b.p.m., and 94% oxygen saturation on room air. Cardiorespiratory examination revealed crackles at the lung bases.

He was a non-smoker with a history of untreated hypertension and dyslipidaemia. The differential diagnoses included acute coronary syndrome, aortic dissection, and pulmonary embolism.

**Figure 1:**
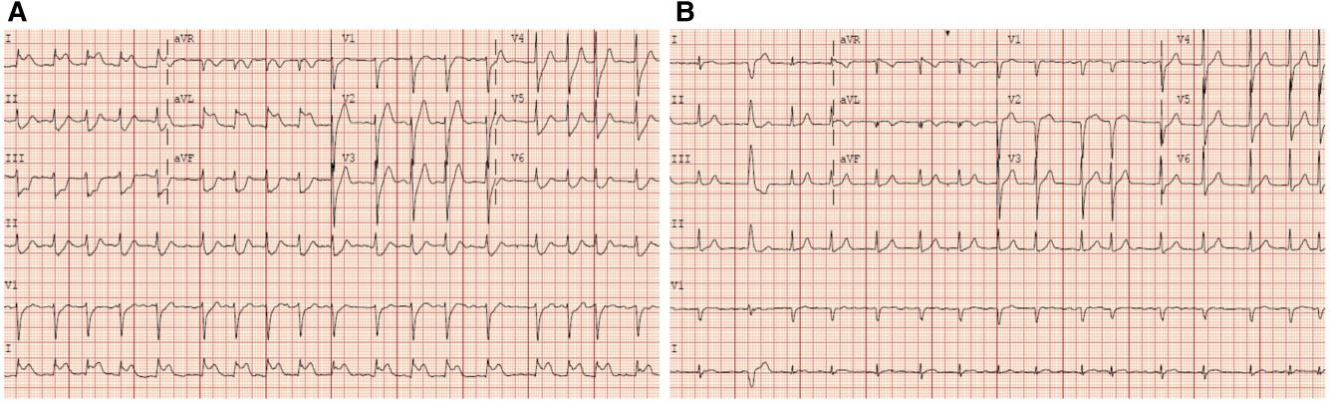
Electrocardiogram. (*A*) Electrocardiogram at presentation showing atrial fibrillation with 3 mm of ST elevation in the leads I and aVL and reciprocal ST depression in leads II, III, aVF, and V2–V6. (*B*) Electrocardiogram post-primary percutaneous coronary intervention showing resolution of ST elevation and ST depression.

Electrocardiogram (ECG) demonstrated atrial fibrillation (AF) with a ventricular rate of 108 b.p.m., 3 mm of ST elevation in the high lateral leads, and reciprocal ST depression in the anterior and inferior leads (*Figure 1A*). Laboratory tests at admission revealed a high-sensitivity troponin-I of 25 ng/L [reference range (RR) < 4 ng/L], haemoglobin of 16.7 g/dL (RR 13.5–17.7 g/dL), white cell count of 22.5 K/µL (RR 4–11 K/µL), platelet count of 245 K/µL (RR 150–400 K/µL), and creatinine of 1.12 mg/dL (RR 0.67–1.17 mg/dL).

**Figure 2:**
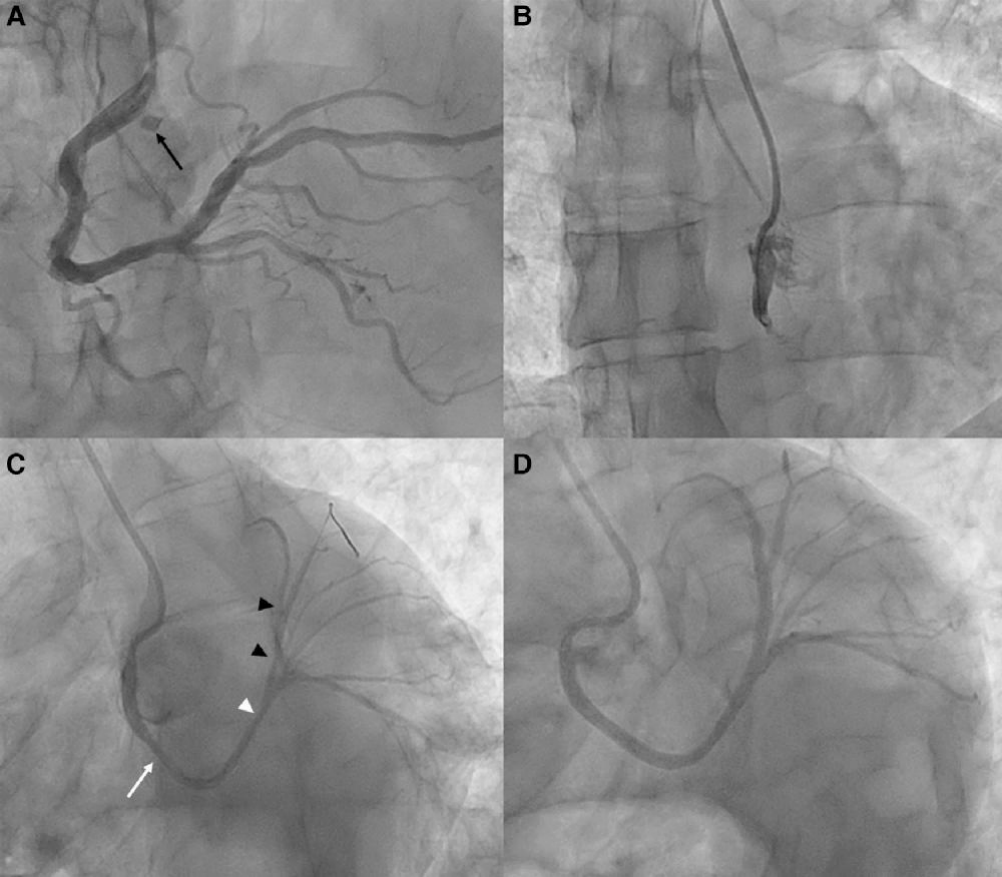
Coronary angiography and percutaneous coronary intervention. (*A*) Selective right coronary artery angiography showing a large, dominant right coronary artery with minor disease and a persistent dye stain (arrow) in the right coronary cusp, indicating an occluded anomalous left main coronary artery. (*B*) Successful cannulation of the occluded anomalous left main coronary artery using an Amplatz Left 0.75 guide catheter via right radial access. (*C*) Restoration of flow after balloon dilatation of the proximal left main coronary artery, revealing residual lesions in the proximal left main coronary artery (white arrow), distal left main coronary artery (white arrowhead), and proximal to mid left anterior descending artery (black arrowheads). (*D*) Final angiography at the end of the case demonstrating good angiographic result with Thrombolysis in Myocardial Infarction 3 flow.

The patient was diagnosed with a Killip class II STEMI. He was loaded with 325 mg of aspirin, 180 mg of ticagrelor, and 4000 units of intravenous (i.v.) heparin before being transferred to the catheter laboratory for primary PCI. Right radial arterial access was obtained, and the right coronary artery (RCA) was engaged using a Judkins Right 4 catheter. This demonstrated minor diffuse disease in a large dominant RCA. We then briefly attempted to engage the LMCA using an Extra Backup Left 3.5 guide catheter without success. Upon review of the RCA angiogram, a persistent dye stain was noted in the right coronary cusp near the origin of the RCA (*Figure 2A*, Supplementary material online, *Video S1A*). Using an Amplatz Left 0.75 guide catheter, we successfully engaged the LMCA and revealed a total occlusion in its proximal segment (*Figure 2B*, Supplementary material online, *Video S1B*). The occlusion was wired and predilated with a 2.5 × 15 mm semi-compliant balloon, which restored flow and revealed a residual 50% stenosis of the proximal LMCA, 70% stenosis of the distal LMCA, and 70% stenosis of a long segment of the left anterior descending artery (LAD; *Figure 2C*, Supplementary material online, *Video S1C*). The proximal LMCA was stented with a 3 × 22 mm drug-eluting stent (DES) and post-dilated with a 3.25 × 20 mm non-compliant (NC) balloon. The LAD and distal LMCA lesions were stented with a 2.5 × 38 mm DES and post-dilated with a 3.25 × 20 mm NC balloon. A distal stent edge dissection at the LAD leading to compromised flow was noted and treated with an overlapping 2.25 × 15 mm DES. This resulted in a good angiographic result with Thrombolysis in Myocardial Infarction (TIMI) grade 3 flow (*Figure 2D*, Supplementary material online, *Video S1D*), as well as ST-segment resolution on the ECG (*Figure 1B*).

After the procedure, the patient was treated with aspirin 81 mg daily, clopidogrel 75 mg daily after a loading dose of 300 mg, atorvastatin 80 mg daily, and i.v. heparin to maintain a partial thromboplastin time (PTT) of 60–80 s. He developed heart failure on Day 1 post procedure and was started on i.v. furosemide 40 mg twice daily, losartan 12.5 mg daily, and metoprolol 12.5 mg four times daily. Weight-based heparin dosing was given at 800 units/h initially, which failed to achieve the target PTT. The infusion rate was progressively increased to 1800 units/h by Day 5, eventually achieving the target PTT of >60 s.

**Figure 3:**
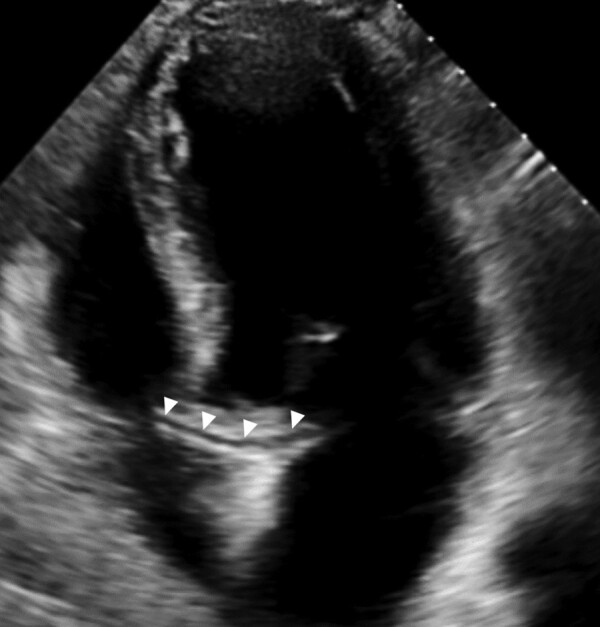
Transthoracic echocardiogram. Transthoracic echocardiogram demonstrating the retroaortic anomalous coronary sign in the apical four-chamber view (arrowheads).

**Figure 4:**
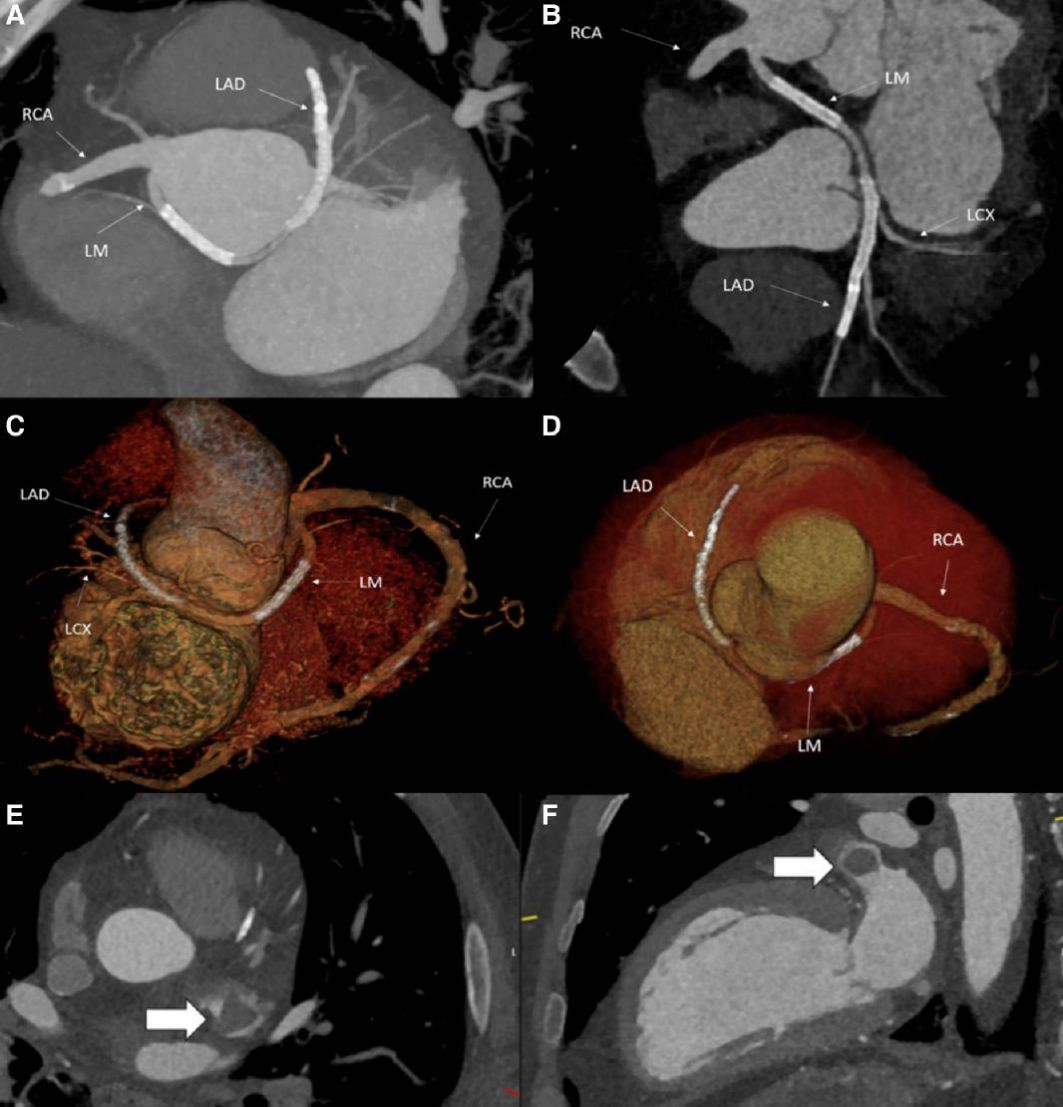
Computed tomography angiography. (*A* and *B*) Multi-planar reconstruction images showing origin of the anomalous left main coronary artery from the right coronary sinus, with stents in the left main coronary artery and left anterior descending artery, and the absence of intramural course, acute angulated take-off, or slit-like narrowing. (*C* and *D*) 3D volume rendering images showing retroaortic course of the left main coronary artery. (*E* and *F*) Large filling defect (indicated by block arrows) in the left atrial appendage indicative of a thrombus.

Transthoracic echocardiogram (TTE) demonstrated mild left ventricular systolic impairment (ejection fraction of 45%) with septal, anterior, and lateral wall hypokinesis. The right ventricle was normal in size and function. Both atria were of normal size, and no significant valvular disease was identified. There was no pericardial effusion. The retroaortic anomalous coronary sign was seen in the apical four-chamber view (*Figure 3*).^4^ Computed tomography angiography (CTA) confirmed anomalous origin of the LMCA from the right coronary cusp with a retroaortic course, without additional high-risk features of intramural course, angulated take-off, or slit-like narrowing (*Figure 4A–D*). Additionally, a left atrial appendage (LAA) thrombus was identified (*Figure 4E* and *F*). On Day 5, he developed abdominal pain, haematuria, and fever. Laboratory testing demonstrated a haemoglobin of 15.7 g/dL, white cell count of 17.1 K/µL, platelet count of 262 K/µL, and creatinine of 1.51 mg/dL. Antithrombin III level was normal at 95% (RR: 83–128%), and C-reactive protein and fibrinogen were elevated at 16.3 mg/dL (RR: <0.5 mg/dL) and 946 mg/dL (RR: 234–395 mg/dL), respectively. A computed tomography (CT) chest and abdomen demonstrated acute bilateral segmental pulmonary emboli and embolic occlusion of the right renal artery with associated infarction. Bilateral lower limb duplex revealed a non-occlusive thrombus in the right distal femoral vein. Losartan was held due to acute kidney injury.

Given his presentation with extensive combined arterial and venous thromboembolism (VTE), additional thrombophilia workup was performed. No family history of VTE was identified. Testing for paroxysmal nocturnal haemoglobinuria, myeloproliferative neoplasms, disseminated intravascular coagulation, and heparin-induced thrombocytopenia was negative. Anticardiolipin antibodies and beta-2 glycoprotein were negative, but lupus anticoagulant results were indeterminate due to concomitant treatment with heparin. A TTE with agitated saline test was negative and ruled out a right to left interatrial shunt. The patient had a nasopharyngeal swab for COVID-19 on Day 5 that returned negative. His creatinine peaked at 2.38 mg/dL on Day 8. Valsartan 40 mg daily was started on Day 10. Spironolactone 12.5 mg twice daily and empagliflozin 10 mg daily were started on Day 11. He was transitioned from i.v. heparin to warfarin on Day 13, with bridging enoxaparin 80 mg twice daily until the international normalized ratio was therapeutic between a target level of 2–3. He made a full recovery and was discharged on Day 14 in a stable condition.

**Figure 5:**
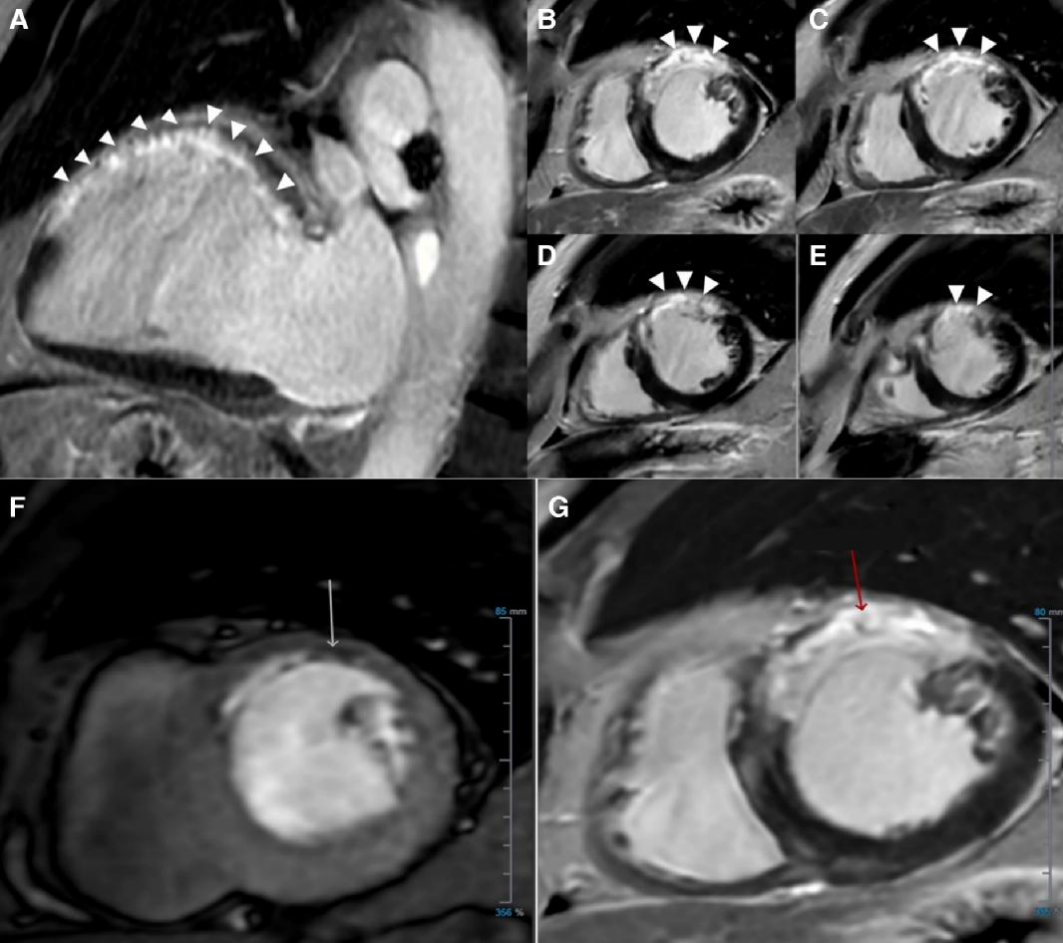
Cardiac magnetic resonance. (*A*) Two-chamber long-axis view showing extensive late gadolinium enhancement (arrowheads) in the anterior wall with wall thinning consistent with transmural infarction. (*B*–*E*) Short-axis view showing late gadolinium enhancement (white arrowheads) in the anterior, anteroseptal, and anterolateral walls consistent with non-viable myocardium in the left anterior descending artery territory. (*F*) Subendocardial perfusion defect in the anterior wall (white arrow) correlating to the (*G*) area of infarction (red arrow), with no reversible ischaemia seen.

At 2-month follow-up, he was asymptomatic with no angina or heart failure. His creatinine improved to a new baseline of 1.25 mg/dL. Repeat testing for lupus anticoagulant was negative. Cardiac magnetic resonance (CMR) demonstrated a left ventricular ejection fraction of 40% and extensive late gadolinium enhancement in the anterior and basal to mid-anteroseptal walls, with sparing of the apex due to supply from the posterior descending branch of the large RCA (*Figure 5A–E*). No reversible perfusion was identified on regadenoson stress (*Figure 5F* and *G*), and there was resolution of the LAA thrombus. Due to the lack of symptoms, ischaemia, and high-risk imaging features, as well as non-viable LAD territory, the decision was made to pursue medical management rather than surgical re-implantation of the anomalous LMCA, in keeping with recommendations from the 2020 European Society of Cardiology Guidelines for the management of adult congenital heart disease.^1^ The patient was offered conversion from warfarin to a direct oral anticoagulant for which he declined. As this was considered a provoked VTE event, he was planned for a repeat chest CTA and lower limb ultrasound 6 months post discharge, after which a patient-centred discussion will be held regarding potential discontinuation of anticoagulation.

## Discussion

In their classic study of 126 595 patients undergoing coronary angiography, Yamanaka and Hobbs^5^ identified an anomalous origin of the RCA from the left coronary sinus as the commonest subtype (0.11%) of AAOCA, followed by LAD from the right coronary sinus (0.03%) and LMCA from the right coronary sinus (0.02%). Although rare in the general adult population, AAOCAs are over-represented in young patients with sudden cardiac death (SCD). In one such study of SCD in 134 young competitive athletes, an AAOCA was the second most common cause identified after hypertrophic cardiomyopathy.^6^ In a separate study of 6.3 million young military recruits, SCD occurred in 64 recruits, 21 (33%) of which had an AAOCA on autopsy.^7^ Because of its rarity, there are no guideline recommendations for screening of this condition in the general population, although either cardiac CT or coronary angiography is recommended as a screening test for coronary anomalies in survivors of cardiac arrest.^8^

After arising from the opposite sinus, an anomalous coronary artery can take an interarterial, retroaortic, prepulmonic, or subpulmonic course, which largely determines the prognosis of the condition.^9^ The interarterial course is the most common subtype associated with SCD in young athletes, presumably secondary to exercise-induced expansion of the great vessels and compression of the anomalous coronary artery, resulting in ischaemia and precipitation of fatal ventricular arrhythmias.^10^ Additional high-risk features include intramural course of the artery, acute angulated take-off of the ostium, and slit-like narrowing of the proximal vessel, all of which may further aggravate coronary compression and ischaemia.^11^

Anomalous aortic origin of a coronary artery can be diagnosed using a variety of imaging techniques including echocardiography, CTA, magnetic resonance angiography (MRA), and invasive coronary angiography. The 2020 European Society of Cardiology Guidelines recommend the use of CTA to diagnose AAOCA, due to its non-invasive nature and ability to assess high-risk features.^1^ Surgical coronary revascularization is recommended for patients with AAOCA and objective evidence of myocardial ischaemia as well as asymptomatic anomalous LMCA with no ischaemia but high-risk anatomy; surgery can also be considered for patients with anomalous LMCA in the absence of ischaemia or high-risk features if they present at age <35 years.^1^

Outside of SCD or aborted cardiac arrest, another clinical scenario one may encounter is the incidental finding of an AAOCA during coronary angiography. The presence of this congenital abnormality increases the technical challenge and risks of procedural complications.^2,3^ To date, there have been rare reports of AAOCA encountered during STEMI,^3,12^ and ours is the first reported case of an occluded anomalous LMCA arising from the right coronary sinus. Given the large myocardial territory at risk, prompt revascularization was critical to achieving a good outcome in this case. The strengths of our approach included quick recognition of the presence of anomalous origin of the LMCA from the persistent dye stain in the right coronary sinus, and the selection of an appropriate guide catheter to facilitate selective engagement of the anomalous coronary artery. It is often challenging to perform PCI in patients with AAOCA, especially with the added time pressure inherent during a STEMI. The main limitation was that we did not perform intracoronary imaging to delineate whether the mechanism of the LMCA occlusion was secondary to embolization from the LAA thrombus or plaque rupture from underlying atherosclerosis. However, there were visible stenoses in the LMCA and LAD on the angiogram after balloon predilation; this coupled with the finding of mild coronary atheroma on the post-PCI CTA points to plaque rupture as the most likely culprit in this case.

Our patient developed extensive VTE and arterial thrombosis in the context of heparin resistance, requiring larger than usual doses of heparin before a therapeutic PTT was achieved by Day 5. Given the normal antithrombin III level in conjunction with elevated C-reactive protein and fibrinogen, the underlying mechanism for heparin resistance in this case was attributed to an acute inflammatory state, which is known to cause elevated levels of factor VIII and fibrinogen, resulting in shortening of the PTT.^13^ The arterial thrombosis was explained by embolization of the LAA thrombus in the context of AF, while VTE was likely secondary to the development of heart failure, which has been estimated to increase the risk of VTE three-fold.^14^

This case highlights the importance for operators to be cognizant of rare congenital coronary artery abnormalities that may be encountered during primary PCI, and to utilize clues present on the angiogram to tailor their approach to the procedure.

## Conclusion

An occluded coronary artery with anomalous origin can be rarely encountered in patients presenting with STEMI. Timely recognition of this congenital anomaly is critical to achieving rapid reperfusion and a good clinical outcome.

## Supplementary Material

ytae192_Supplementary_Data

## Data Availability

The data underlying this article will be shared upon reasonable request to the corresponding author.

## Lead author biography

Dr Christopher Wong is an interventional cardiology fellow at Stanford University Medical Center. He obtained his undergraduate medical degree from the University of Adelaide and his PhD from the University of Sydney. His research is primarily focused on coronary physiology and interventions.

## Notes

**Consent:** The authors confirm that written consent for submission and publication of this case report, including images and associated text, has been obtained from the patient in line with COPE guidance.

**Funding:** None declared.
