# Supplementary material for: A case report of successful primary percutaneous coronary intervention to an occluded anomalous left main coronary artery arising from the right coronary sinus
Source: Eur Heart J Case Rep. 2024 Apr 15;8(4):ytae192. doi: 10.1093/ehjcr/ytae192 (PMC11044188; doi:10.1093/ehjcr/ytae192)
Supplement: ytae192_Supplementary_Data [file ytae192_supplementary_data.zip › Supplementary Appendix.docx]

**Supplementary Video**

Supplementary video 1: **(A)** Cranial projection during selective RCA angiography, showing a large dominant RCA and a persistent dye stain in the right coronary sinus representing the occluded anomalous LMCA; **(B)** Anteroposterior projection demonstrating successful selective cannulation of the occluded LMCA with an Amplatz Left 0.75 guide catheter; **(C)** Caudal left anterior oblique projection showing restoration of flow in the LMCA after balloon angioplasty, with residual stenoses in the LMCA and LAD; **(D)** Final angiographic result in the caudal left anterior oblique projection showing TIMI 3 flow after LMCA and LAD stenting.
